# Policy design for territorial equity in multi‐level and multi‐sectoral political systems: Comparing health and education strategies

**DOI:** 10.1111/rsp3.12466

**Published:** 2021-09-21

**Authors:** Paul Cairney, Sean Kippin, Emily St Denny, Heather Mitchell

**Affiliations:** ^1^ University of Stirling – History, Heritage, and Politics UK; ^2^ Department of Political Science University of Copenhagen Denmark

**Keywords:** education equity, Health in All Policies, spatial justice, territorial inequalities

## Abstract

The EU has many plans to foster equity and spatial justice. However, each has separate reference points, and it is difficult to find an overall vision. To demonstrate, we analyse two sectoral strategies to identify their implications for spatial justice strategies. Education focuses on early investment and public service reform. Health prioritises intersectoral action to address the ‘social determinants’ beyond the control of health services. Both warn against equating territorial cohesion or spatial justice with equal access to public services. These findings could inform European Commission strategy, but it tends to respond with renewed rhetoric rather than reconsidering its approach.

## Resumen

La UE tiene muchos planes para fomentar la equidad y la justicia espacial. Sin embargo, cada uno tiene puntos de referencia distintos, y es difícil encontrar una visión global. Para demostrarlo, este estudio analizó dos estrategias sectoriales para identificar sus implicaciones en las estrategias de justicia espacial. La educación se centra en la inversión temprana y la reforma de los servicios públicos. La salud prioriza las acciones intersectoriales para abordar los ‘determinantes sociales’ más allá del control de los servicios sanitarios. Ambos sectores advierten del peligro de equiparar la cohesión territorial o la justicia espacial con la igualdad de acceso a los servicios públicos. Estos hallazgos podrían informar la estrategia de la Comisión Europea, pero ésta tiende a responder con una retórica renovada en lugar de reconsiderar su enfoque.

## 抄録

EUには平等と空間的正義を促進する多くの計画がある。しかし、計画はそれぞれ基準点が異なり、全体像を捉えるのは困難である。そこで、2つのセクターのストラテジーを分析し、空間的正義のストラテジーに対する意義を確認して実証を試みた。教育セクターは、早期投資と公共サービスの改革に焦点を合わせている。保健医療セクターは、保健サービスの範囲を超えた「社会的決定要因」に対処するためにセクター間での行動を優先している。両者は、領土的結束 (Territorial Cohesion)や空間的正義と公共サービスへの平等なアクセスとを同一視することに対して警告を発している。以上の結果は欧州委員会のストラテジーに影響を与える可能性があるが、欧州委員会はアプローチを見直すのではなく、新たなレトリックを用いて対応する傾向がある。

## 1 INTRODUCTION

The European Union (EU) is committed to achieving greater equity within and across member states. This broad concept describes reducing unfair inequalities, and connects to similar values such as justice. As such, the EU seeks to foster equity in multiple connected ways, including in *sectors*, via health and education equity policy, and *regions*, via territorial cohesion policy. The IMAJINE (Integrative Mechanisms for Addressing Spatial Justice and Territorial Inequalities in Europe) project seeks to understand and support this pursuit of spatial justice in the EU. The aim of this paper is to establish how intersectoral equity initiatives (and multi‐level policy‐making) contribute to policies designed to reduce territorial inequalities.

However, this aim is easier said than done, since there is no single coherent overarching approach to equity or justice in theory or practice. Rather, the meaning of key concepts is subject to continuous contestation and change, in four main ways. First, while ‘spatial justice’ draws attention to the geographic aspects of socio‐economic inequalities, it remains ‘ill‐defined’ in relation to other key aims (‘social justice’ or ‘social equity’) and means (such as ‘procedural’ or ‘distributive’ justice) (Madanipour et al., 2021: 3). Second, there is contestation to prioritise some forms of inequality over others (such as in relation to class, gender, race, and migration status), determine the cause of the problem (a structural problem to be solved by the state, or an individual problem to be solved privately) and establish what amount of inequality is fair. Third, the responsibility for making sense of and delivering such aims is shared across *multiple levels of government* (including EU, member states, regional and local). Fourth, separate equity initiatives exist in *multiple sectors* even when their stated aims are intersectoral (including economic, rural, health, education, and gender mainstreaming strategies).

EU policy‐making reflects this ambiguity and contestation. Approaches to equity and spatial justice have changed direction over time (Weckroth & Moisio, 2020), and they are taken forward in governments and sectors with their own history, assumptions, frames of reference, policy networks and impacts on public services. General aims – such as equity and spatial justice – are interconnected in theory, but their relationship is unclear in practice. We may *hope* that they combine to produce a coherent long‐term approach, but find in practice that many approaches are contradictory. To understand and foster spatial justice therefore requires us to make sense of it on its own terms *and* in relation to the EU’s many different ways to pursue equity. To that end, we identify the unclear relationship between multiple equity initiatives and relate this problem to the study of spatial justice.

To do so, we build on two key insights from current spatial justice research. First, *to highlight the connection between contested concepts and vague policies*. There is high commitment to general aims to reduce territorial inequalities, backed by terms such as territorial cohesion. National, regional and local governments use a similar language to support similar aims. However, there is little agreement about what spatial justice ultimately means, and how to cooperate to achieve it. Studies of EU policy identify contestation, shifting policy aims, limited progress and the search for alternatives. There are new debates about key terms (what does the pursuit of spatial justice mean, and is it a useful alterative to cohesion?) and who should take responsibility for making sense of them (the EU or regions?).

For example, Weckroth and Moisio (2020: 183–6; 190) examine two decades of EU ‘territorial cohesion’ policy: (1) identifying its general commitment to address ‘uneven geographical development’ but also its ‘peculiar, elusive and contested’ nature; and, (2) tracing its intellectual development, from a macroeconomic focus on economic growth and regional gross domestic product (GDP) disparities towards improving ‘spatially equal access to services’ and individual perceptions of *regional variations in access to public services* (2020: 187). Similarly, Madanipour et al. (2021: 9–12) identify a shift of policy over time, from (1) a ‘distributive agenda’, to allocate funding at an EU level, to (2) a ‘procedural agenda’ that emphasises *multi‐level governance* (MLG), devolving the responsibility to invest within regions, and making sure that ‘sectoral policies which have a spatial impact’ and ‘regional policy’ are ‘more coherent’. Further, Jones et al. (2020: 894–6) describe: (1) pressure on EU cohesion policy resulting from its ‘dubious economic effectiveness’ (its own measures suggest that regional disparities are increasing) and failure to counter ‘growing political and philosophical critiques of the very ideals of furthering European integration’; and, (2) the push for new ways of thinking, based on the autonomy of regions to define and promote their own spatial justice agendas (rather than being seen as perennial losers in a race to the top).

Second, *to highlight the impact of multi‐level and multi‐sectoral policy‐making on ambiguous policy agendas*. In particular, while MLG is often proposed as a way to foster policy coherence, it may contribute to the opposite. Cairney et al. (2020) identify what happens when multiple levels of government seek to make sense of the same vague multi‐level and intersectoral agenda. Their case study highlights the perception within governments that gender mainstreaming policy lacks coherence. In this context, policy incoherence means ‘a lack of joined‐up government that contributes to a confusing mix of policy instruments’ which ‘contributes to a major gap between expectations and policy outcomes’ (2020: 2). They provide four relevant reasons for this appearance of incoherence: (1) if ‘mainstreaming’ relates to all policy in all relevant governments, it is difficult to identify a manageable number of complementary initiatives; (2) many governments share responsibility for the policy instruments that contribute to policy; (3) individual governments deal with unmanageability by breaking responsibility into sectors and subsectors, each with their own logics and silos; and (4) they encourage decentralised and ‘co‐productive’ ways to define policy agendas in different contexts (2020: 7–11). The overall effect is low clarity on what to do and who should do it.

From this research, we extract three interconnected arguments to inform our study of spatial justice and intersectoral equity policies. First, policy ambiguity has favourable and unfavourable consequences: it is essential to generate initial policy agreement, but then it becomes an obstacle to policy delivery. If a concept means everything, it also means nothing (Wildavsky, 1973), prompting superficial agreement on little more than a vague strategy. Second, these concepts and aims are often so vague as to become contradictory: some aspects emphasise centralised EU direction to foster uniformity; others emphasise decentralised direction to foster diversity, with the potential to produce policies that contradict EU aims. Third, many ambiguous policy objectives co‐exist without clarity on the extent to which they complement or undermine (a) each other, and (b) government policy as a whole. The latter is particularly important, since a key finding from comparable studies of equity strategies (in social policy) is that they receive high rhetorical commitment in government despite being undermined by many government policies (Cairney & St Denny, 2020). Overall, since equity initiatives’ *practical meaning* may contradict their *rhetorical meaning*, their impact is unclear unless we identify how they interact with each other and routine government business.

We use these insights to investigate the relationship between territorial cohesion and equity policies. First, we describe sectoral equity initiatives in health and education that should be complementary since they stress the need for intersectoral action (Cairney, 2021). Second, we explore how they relate to each other and the idea of spatial justice. We find that they have tenuous connections to each other and pursue different approaches to equity. Further, while the emphasis of territorial cohesion policy on spatially equal access to public services and coherent regional and sectoral policies should have a major impact on those sectors, neither initiative discusses spatial justice substantively, and both challenge the idea that equal access to services fosters equity.

## 2 METHODS

We used the qualitative systematic review method to synthesise empirical data from equity policy research. We published two large (25,000 word) separate reviews of global equity policy research in health and education (see Cairney et al., 2021; Cairney & Kippin, 2021 for a full discussion of methods and coverage). This wider activity allowed us to generate an interdisciplinary approach, using policy theories (from political science) to interpret empirical data produced by health and education researchers and relate it to insights on spatial justice from geographical research. Our project’s general guiding question is: how does equity research use policy theory to understand policy‐making? We then use sub‐questions to guide article inclusion and analysis, including: how do they describe policy‐making and the causes of policy change that are vital to equity strategies, and what transferable lessons do these studies provide for spatial justice? This article focuses on the included articles that provide lessons relevant to EU, state and regional *policy‐making*. The association between policy‐making and policy outcomes (e.g., which countries or regions are the most successful in reducing inequalities) is beyond the scope of this paper, since almost zero articles (included in our reviews) try to measure the discrete impact of policy instruments.

## 3 HEALTH EQUITY STRATEGY: HEALTH IN ALL POLICIES (HIAP)

‘Health in All Policies’ (HiAP) is a global strategy promoted by the World Health Organisation (WHO) and receiving high rhetorical support from most country governments and the EU. In policy documents and research, there is a clear HiAP narrative with the following elements (Cairney et al., 2021: 6–8):

1. Health is a human right to be enjoyed by everyone and supported by governments and international organisations (WHO (World Health Organisation*),* 2014).
2. Yet, health is unequally distributed, and the cause is social rather than biological or caused primarily by individual choices (Whitehead & Dahlgren, 2006: 4).
3. To promote health equity, we need to focus on the ‘social determinants’ of unfair health inequalities, caused by inequalities in ‘social and economic factors, including employment opportunities, the law and the justice systems, education, housing, neighborhood environments, and transportation’ and ‘too often associated with a person's socioeconomic status, race, ethnicity, gender, religion, sexual identity, or disability’ (Bliss et al., 2015: S88).
4. Most of the advocated policy measures are not health sector focused. Equal access to healthcare is not the primary focus, since the most effective population health measures are ‘upstream’, ‘aimed at fundamental social and economic reform … for the redistribution of wealth, power, opportunities, decision‐making capacities, and other resources’ (Shankardass et al., 2011: 29).
5. Responsibility for most measures – to redistribute income, improve education, housing and transport, reduce discrimination and violence, and improve social, economic and physical environments – is not in the gift of health departments.
6. Effective policy‐making requires collaboration across all sectors of government, and with stakeholders and citizens outside of government (Cairney et al., 2021: 8–10).
7. Long‐term success requires high and enduring political support. It may help produce a high‐level strategy, cut through ‘administrative silos’ (Carey and Crammond, 2015), generate support for new measures to institutionalise health equity procedures (such as the application of Health Impact Assessments to non‐health policies) and reduce implementation problems.
8. Policy failure involves a drift from HiAP’s focus on state intervention to address the social determinants of population health, towards ‘neoliberalism’ and ‘lifestyle drift’ in which there is a preference for non‐state action and a return to focusing on individual choices (De Leeuw & Clavier, 2011: 237–40).

In that context, there is high *potential* to relate HiAP to spatial justice policies. Examples include:

- ‘Healthy cities’, where local policy‐makers commit to intersectoral collaboration to improve population health
- The idea of a ‘postcode lottery’ in which population health and the availability and quality of healthcare varies spatially
- The motivation of national central governments to centralise policy‐making (to reduce a postcode lottery) or decentralise (to address territorial demands for autonomy and/or to tailor services to local populations)
- The (limited) impact of subnational government autonomy on health spending and outcomes (Cairney et al., 2022; Costa‐Font & Greer, 2012; De Leeuw & Simos, 2017)

However, most HiAP research – in EU, country and subnational studies – focuses more narrowly on the contrast between high rhetorical commitment to policy change versus low follow‐through. Godziewski (2020a: 1307) notes that HiAP was embraced rhetorically during Finland’s EU presidency in 2006, and ‘is regularly referred to by the Commission, but has not yet been implemented as an overarching political vision’. While the focus on non‐health sectors gives the EU a new impetus to act (its health sector powers are relatively limited), EU governance tends towards a ‘neoliberal rationality’ and giving greater priority to economic over social policy, which limits the substantive changes to policy and policy‐making suggested by HiAP (2020a: 1308–13).

Godziewski (2020b: 2) relates this obstacle to the ambiguity of HiAP and its advocates’ non‐confrontational approach to policy change in other sectors, which make ‘this policy agenda particularly prone to being reinterpreted, and ultimately watered down’. Advocates avoided giving the sense that HiAP required a radical shift in policy, which made it attractive but also vulnerable to being interpreted as little more than a means to work collaboratively (2020b: 2–3). Indeed, when the European Commission treated HiAP as a tool for stakeholder collaboration, it represented a way to involve non‐health actors in health policy. It included food and drink companies that public health actors treat with suspicion (2020b: 6). This move helps explain the EU’s ‘lifestyle drift’ in the case study of diet policy (2020b: 8). Further, such examples show that HiAP was only able to become a part of ‘orthodox policymaking spaces’ if its ‘political, normative essence is toned down so as to not fundamentally challenge the status quo’ (2020b: 2–3).

Cairney et al.’s (2021: 15–18) review suggests that country and local experiences of HiAP demonstrate similar obstacles. For example, Finland is highly committed to HiAP, and aided by a social democratic welfare state, meaningful decentralised public health action and a ‘culture of collaboration and societal values’ (Kokkinen et al., 2019; Puska & Stahl, 2010; Ståhl, 2018). Yet, other central government policies undermined this approach, including a combination of market‐based reforms (some of which were prompted by EU accession) and austerity measures, while centrally driven HiAP reforms were not conducive to local innovation, and there is little evidence for policy change in many municipal governments (Ståhl, 2018: 43). There are similar experiences in Norway (outside of the EU), in which the welfare state and national legislation is conducive to progress, but most municipal governments (tasked with the coordination of detailed policy changes) struggle to translate general enthusiasm into coordinated action across sectors (Fosse & Helgesen, 2017; Hagen et al., 2018; Synnevåg et al., 2018). Similar research on Denmark, Sweden and the Netherlands reports a high‐but‐vague national commitment to HiAP, coupled with limited and variable progress among municipal governments and a tendency for intersectoral action to lead to policies focusing on individual lifestyles (Holt et al., 2018; Peters et al., 2016; Scheele et al., 2018; Storm et al., 2014).

## 4 EDUCATION EQUITY STRATEGIES

Compared with HiAP, education research describes equity policy as relatively contested. There are more debates on fair or acceptable inequalities in relation to socio‐economic background, regarding:

1. The difference between *horizontal equity* (equal provision regardless of background) and *vertical equity* (unequal provision to mitigate against unequal backgrounds).
2. Equity based on *‘merit’*, which contributes to ‘severe inequalities’ in outcomes, a *threshold of attainment*, which maintains ‘relative advantages’, and *justice*, to redistribute resources to reduce geographical inequalities or pursue equality of outcomes such as attainment.
3. The extent to which the state should take responsibility for education inequalities (Gilead, 2019: 439).

These debates inform competing international agendas (Cairney & Kippin, 2021). One is a *social justice* narrative that represents education’s closest equivalent to HiAP: initiatives promoted by the United Nations Educational, Scientific, and Cultural Organization (UNESCO, 2021a, 2021b, 2021c) to treat education as a human right, challenge marginalisation in relation to ‘sex, ethnic/social origin, language, religion, nationality, economic condition, ability’, and foster early years education; and the UN Sustainable Development Goal 4 to ‘Ensure inclusive and equitable quality education and promote lifelong learning opportunities for all’. Another is an *economic (‘neoliberal’) narrative* supported by organisations such as the World Bank, identifying the role of education to boost human capital and economic competitiveness in a global knowledge economy, promoting a combination of non‐state solutions to equity (such as incentive schemes to attend ‘high quality’ schools) and ‘new public management’ measures to encourage improvements to education system performance and school performance (Faul, 2014; Klees & Qargha, 2014).

Most articles in Cairney and Kippin’s (2021) review suggest that the economic narrative dominates international and domestic policy agendas. While there is high support to address unfair inequalities, it relates primarily to minimum standards, including global access to primary education, equal access to high‐quality education within each system, and for all students to achieve a threshold of attainment. Further, the Organisation for Economic Co‐operation and Development (OECD) enjoys high global agenda setting influence, and identifies the ability of education systems to mitigate against socio‐economic inequalities through: (1) better school performance and (2) the redistribution of spending (a) to address regional disparities and (b) in favour of early years over higher education (Field et al., 2007; OECD, 2008, 2012). Overall, the international agenda focuses on measuring system and school performance as a means to boost economies *and* reduce inequalities, but with the former taking priority. Equity is primarily the equal opportunity to access public services such as schools, aided by some commitment to measures to address regional imbalances, with equitable outcomes relating to minimum standards.

As with HiAP, there is high *potential* to connect education equity to spatial policy‐making, in relation to:

1. Competing motives to decentralise responsibility to allow local governments and schools to tailor services to local contexts, *and* centralise performance management and national testing to improve standards and accountability.
2. The ability of some regional or devolved governments to design and deliver their own education equity policies and seek distinctive outcomes (Cairney et al., 2022).

However, most EU, country and regional studies focus on the alleged tendency for ‘neoliberal’ policies to dominate and undermine ‘social justice’ approaches, and for national performance management and accountability measures to undermine local variations.

Work by the European Commission (2006: 2–4) reflects this economics‐driven understanding of education equity, albeit with some recognition of the wider determinants of outcomes and need for intersectoral action. It makes the case that ‘high quality education and training systems’ should be ‘efficient and equitable’ (2006: 2). *Efficiency* relates to the best time to invest in human capital. It reproduces the famous ‘Heckman curve’ to (1) support the earliest possible investment in high‐quality education, particularly for ‘children from low socio‐economic background’, and (2) criticise the inequity of higher education investment, since participation is limited and the tax system does not offset their higher earnings (2006: 4–5; see also Woessmann & Schuetz, 2006: 10–14; Vandenbroeck, 2007). *Equity* relates to two elements:

1. *Human capital*. Low investment in education reforms now means lower productivity later, since existing systems exacerbate inequalities in relation to socio‐economic challenges, including globalisation, digital economies, ageing, migration, and labour market changes (European Commission, 2006: 3).
2. *The governance of schools*. It notes that many member states have decentralised key aspects of school governance, which requires a parallel commitment to centralised accountability mechanisms and other measures – such as standardised formal examinations to monitor attainment outcomes, evaluations of school progress, and motivating the most experience and skilled teachers ‘into the most challenging schools’– to ‘avoid the potentially inequitable local consequences of decentralised decisions’ (2006: 6–7).

Hippe et al.’s (2016: 5) update to Commission work pays more attention to gender, immigration and regional disparities. However, they largely maintain the original focus on school quality and performance as the main vehicle for equity, recommending that school systems foster ‘autonomy coupled with accountability’ and ‘competition sponsored by public funding’, while teachers receive ‘rewards for their performance’. One rationale for this Commission focus is the political context in which redistributional policies would address the relationship between income and education attainment, but remain ‘controversial’ and less amenable to public policy, while policies to foster equal ‘opportunities’ to access high‐quality education would be more popular (2016: 7; see also Eurydice, 2020). There is also some discussion of the role of private provision and incentive schemes (such as vouchers to facilitate school choice), while acknowledging that competition may boost high attainment at the expense of equal attainment (Hippe et al.*,* 2016: 9; 14–20; drawing on Braga et al., 2013: 51–4; see also Woessmann & Schuetz, 2006: 16; 20; 31; Demeuse et al., 2007: iii‐v).

Country and regional studies of education policy describe a dynamic similar to HiAP, in which a rhetorical focus on the pursuit of social justice and education equity becomes undermined by a wider economic and performance management agenda (Cairney & Kippin, 2021; Hajisoteriou & Angelides, 2020). Again, Nordic experiences are the most common sources of best‐case scenarios and cautionary tales, with multiple studies describing the threat of neoliberalism to (a) social democratic values built on trust and social capital and (b) education systems that foster comprehensive and non‐selective schools. Finland represents the system most resilient to such forces (Chong, 2018: 502), with decentralisation taking place in the context of comprehensive schooling and no tradition of ‘mandatory national testing … school inspections and school league tables’ (Varjo et al., 2018: 486). In contrast, Swedish governments encouraged a larger private sector, with a marked spread by geography and class. They fostered school choice via vouchers for students, which contributed to competition between state and independent schools (2018: 486–9). There is also evidence of rural student commutes to cities but not vice versa, prompting some rural schools to sell themselves as more welcoming to local immigrant populations (2018: 490–1).

Power and Frandji (2010: 394) describe similar tendencies towards performance management in France and England, while Chapman and Ainscow (2019: 899) highlight the tensions between centralisation and decentralisation when education systems in England, Scotland and Wales combine a top‐down mandate with bottom‐up delivery. Further, studies of other countries highlight a tendency for this dominant focus on economic competitiveness and performance management to undermine wider social justice agendas. In Denmark, Engsig and Johnstone (2015: 472) describe the challenge of a neoliberal approach to its formerly dominant focus on social inclusion (see also Pettersson et al., 2017 on Norway). In Cyprus, a general focus on equal access to schools, combined with vagueness in government aims (to respect ‘diversity and cultural, linguistic and religious pluralism’), ensures that schools reproduce ‘cultural domination, non‐recognition and disrespect’ and do not adapt their equity policies to the social background or cultural practices of marginalised populations: ‘it appears that policy‐makers themselves do not value their own policy rhetoric for social justice, thus failing to get schools to take such policy priorities seriously’ (Hajisoteriou & Angelides, 2014: 159; 168).

## 5 DISCUSSION AND CONCLUSION

Our comparison of equity strategies helps to identify contrasting ideas regarding how to define and reduce unfair inequalities within and across the EU, countries and regions. These ideas have direct implications for territorial cohesion policies, following their shift in focus from distributional measures relating to regional GDP towards the pursuit of procedural measures to foster MLG and spatially equal access to public services.

HiAP policy and research argues that health is unequally distributed and the state should intervene to address the ‘social determinants’ of unfair health inequalities. It prioritises intersectoral action since ‘upstream’ policy measures are not in the gift of health departments or services. Further, empirical studies of HiAP provide multiple cautionary tales for equity strategies. First, HiAP advocates propose radical changes, to focus primarily on redistribution and population‐wide measures rather than the reform of health services. As such, it contrasts with a modern territorial cohesion focus on equal access to public services. Second, radical equity agendas tend to be accepted rhetorically because they are vague, allowing non‐committal support with minimal expectation of follow‐through. Indeed, the only sign of HiAP progress relates to non‐radical initiatives that are conducive to fitting in with routine government business. As such, HiAP’s progress relies heavily on existing policy‐making arrangements, with the EU’s greater prioritisation of economic policy making it less conducive to policy progress than countries such as Finland, while Finland’s progress is vulnerable to wider national and supranational ‘neoliberal’ policy programmes. Third, countries with a history of well‐established municipal governments help expose governance dilemmas associated with the scale at which policy should be made and delivered. In theory, HiAP studies describe the right of municipal governments to make sense of HiAP in local areas, aided by cooperation with non‐health actors. In practice, HiAP research views limited progress from the top‐down, bemoaning ‘implementation gaps’ at local levels, and holding local policy‐makers responsible for lifestyle drifts (Cairney et al., 2021: 25–6).

Studies of education equity policy describe more contested narratives in which there are attainment gaps caused by a combination of socio‐economic background, school performance, and individual merit and motivation. Key policy measures relate to early investment in education to boost human and social capital. A common argument has emerged in which early investment in high‐quality early education fosters *efficiency* via a mutually reinforcing process of education at all stages, and *equity* since it has more impact in relation to socioeconomically disadvantaged groups. The more salient debates involve a competition between narratives – neoliberal versus social justice – on equity. The former dominates, focusing on the idea of equal access to educational opportunities: everyone should be able to access a high‐quality education, and competition between schools may boost quality, measured in relation to national tests and international comparisons, aided by measures such as incentive schemes (e.g., school vouchers) and some redistribution of resources across schools or regions. In contrast, researchers supportive of social justice approaches identify the inevitably unequal (and, according to these researchers, inequitable) outcomes from neoliberal systems. Measures sold in the name of education equity exacerbate inequalities via school competition and incentive schemes that produce greater segregation across schools (via selection) and within them (via ‘tracking’ measures), while half‐hearted measures to address multiculturalism and migration do not mitigate against unequal outcomes in relation to race and ethnicity (Schlicht‐Schmälzle & Möller, 2012). There are also national–local tensions, but with more education researcher sympathy for the idea that local leaders can drive equity strategies rather than being responsible for limited progress.

Overall, these comparisons suggest that a rhetorical focus on collaborative policy‐making and equal access to public services is not a panacea for spatial justice and territorial cohesion policies:

1. Both approaches warn against equating spatial justice with equal access to public services: a narrow focus on services ignores the wider social determinants of inequalities, taking attention from the role of redistribution in favour of measuring rather than reducing unequal outcomes.
2. Each approach highlights different tensions in the balance between centralisation and decentralisation, but both conclude that MLG is not necessarily an effective vehicle for cohesive equity policies. Rather, there will always be unresolved debates regarding the scale at which such policies should be made: to *centralise*, to prioritise a sense of common purpose, directed from a single authority; or, to *decentralise*, to prioritise the legitimacy of multiple forms of governance, directed by local policy actors in collaboration with stakeholders and communities to make sense of policy aims (Cairney et al., 2022).
3. Although a focus on equity and justice appears to offer hope for radical policy change, in practice these initiatives become incorporated within routine ways of doing things: HiAP became a vehicle for stakeholder participation, and education a vehicle for public service performance management, rather than a means to encourage distributive justice.

Overall, relating sectoral equity initiatives to spatial justice agendas highlights inconsistent models of policy‐making and expectations for policy. It reinforces the sense that the EU promotes separate equity initiatives without acknowledging if they complement or contradict each other. This action could be unintentional, as a function of policy‐making complexity in which no policy actor has full knowledge or control. Or, it could be intentional, to exploit the ambiguity of equity concepts to make a radical case in rhetoric but actually reinterpret equity initiatives to make them fit routine government business. If so, it would represent one of many measures to ‘depoliticise’ policy‐making by turning highly salient debates on values (how much inequality is acceptable, under what circumstances?) into technical discussions regarding proper processes (stakeholder participation, ‘evidence based’ policy‐making, and performance management). A shift of focus from unequal levels of regional GDP offers a broader and more nuanced perspective on territorial inequalities, but may also represent a way to reduce political attention to those inequalities.
